# Neonatal effect of remifentanil in cesarean section with general anesthesia: A protocol of systematic review and meta-analysis

**DOI:** 10.1097/MD.0000000000020212

**Published:** 2020-05-15

**Authors:** Qi Zhang, Hong-Li Kan, Dong-Xin Wang, Dong-Mei Fu

**Affiliations:** Department of Anesthesiology, Jilin Cancer Hospital, Changchun, China.

**Keywords:** cesarean section, neonatal effect, randomized controlled trials, remifentanil

## Abstract

**Background:**

Although several previous studies have reported the efficacy of remifentanil in cesarean section (CS) under general anesthesia, no study has specifically addressed its neonatal effect (NE) in CS under general anesthesia systematically. Thus, this study will systematically investigate the NE of remifentanil in CS under general anesthesia.

**Methods:**

Electronic databases including MEDLINE, EMBASE, Cochrane Library, Web of Science, Chinese Biomedical Literature Database, and China National Knowledge Infrastructure will be systematically retrieved with the assistance of a specialist librarian to check randomized controlled trials reporting NE in CS under general anesthesia. We will retrieve all electronic databases from their initial time to March 20, 2020 without restrictions of language. All process of study selection, data extraction, and risk of bias evaluation will be carried out by 2 independent authors. We will invite another senior expert to solve the problems that arise between 2 authors. Data will be pooled and analyzed using RevMan V.5.3 software.

**Results:**

Outcomes consist of assessment of neonatal adaptation, requirements for postoperative respiratory support of neonates, systolic and diastolic noninvasive blood pressure, mean blood pressure, heart rate, electrocardiography, umbilical cord blood gas analysis, and adverse events.

**Conclusion:**

This study will present evidence of the NE of remifentanil in CS under general anesthesia. This information may inform benefits of intervention to guide the usage of remifentanil in CS under general anesthesia.

**Study registration:**

INPLASY202040028.

## 1 Introduction

Cesarean section (CS) is one of the most common obstetric surgeries.^[1,2,3,4]^ It helps pregnant women deliver babies because of the dystocia or certain obstetric complications.^[5,6,7]^ Its prevalence increases annually due to the current increased marriage age and socioeconomic status.^[8,9,10,11]^ Participants with CS experience very strong pain intensity.^[12,13]^ Thus, finding a way to relieve pain and minimize complications, effective anesthesia drug choose is very important.^[14,15,16,17]^ A variety of studies found that remifentanil is good choice on neonatal effect (NE) in CS under general anesthesia.^[18,19,20,21,22]^ However, there is no systematic review that specifically assessed the NE in CS under general anesthesia. Therefore, this study will systematically evaluate the NE of remifentanil in CS under general anesthesia.

## 2 Methods

### 2.1 Study registration

This study has been registered on INPLASY202040028. It has been reported following the preferred reporting items for systematic review and meta-analysis protocols.^[23]^

### 2.2 Eligibility criteria

#### 2.2.1 Type of participants

We will include pregnant women (more than 18 years old), who received remifentanil in CS under general anesthesia, irrespective race and educational background.

#### 2.2.2 Type of interventions

We will include all participants who underwent remifentanil in CS under general anesthesia in the experimental group. However, in the control group, all participants can receive any anesthesia intervention except remifentanil.

#### 2.2.3 Type of studies

We will consider all randomized controlled trials that exploring the NE of remifentanil in CS under general anesthesia for inclusion. However, we will not consider any other studies, except randomized controlled trials.

#### 2.2.4 Type of outcome measurements

The primary outcome is the evaluation of neonatal adaptation, as measured using Apgar score or relevant tools. The secondary outcomes are requirements for postoperative respiratory support of neonates, systolic and diastolic noninvasive blood pressure, mean blood pressure, heart rate, electrocardiography, umbilical cord blood gas analysis (such as pulse oximetry, and base excess), and adverse events.

### 2.3 Data sources and search strategy

#### 2.3.1 Electronic searches

In conjunction with a specialist librarian, following electronic databases will be searched from the beginning of each database to March 20, 2020: MEDLINE, EMBASE, Cochrane Library, Web of Science, Chinese Biomedical Literature Database, and China National Knowledge Infrastructure. We will not impose any language and publication status limitations. The example of search strategy for Cochrane Library is built in Table 1. We will also apply similar search strategies to the other electronic databases.

**Table 1:**
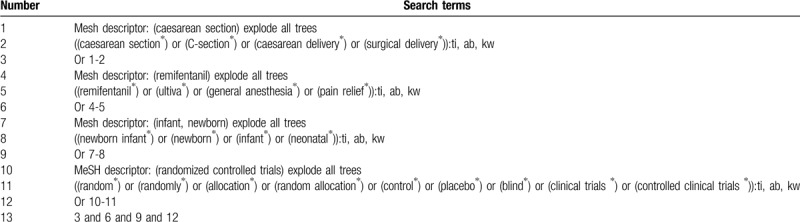
Search strategy for Cochrane Library.

#### 2.3.2 Other resources

In addition, we will also check conference proceedings and reference lists of all included studies.

### 2.4 Data collection and analysis

#### 2.4.1 Selection of studies

Results from all literature citations will be imported into EndNote 7.0, and all duplicated citations will be excluded. Screening of titles and abstracts, and full-text records will be carried out by at least 2 independent authors. Any different views between 2 authors will be solved by consensus or discussion with the help of a third author if needed. The whole process of study selection will be demonstrated using a flowchart. The reasons of all excluded literatures will be recorded at each stage.

#### 2.4.2 Data collection process

Data from all eligible studies will be extracted using a previous designed standardized data collection sheet. At least 2 authors will collect data independently. Any discrepancies between 2 authors will be resolved by another author through discussion to reach a final decision. The extracted information includes study information (such as time of publication, first author, and journal information), study characteristics (such as design, setting, location, and funding information), participant characteristics (such as race, age, sample size, and inclusion and exclusion criteria), intervention and control details (such as dosage, types, and duration), and outcomes (such as primary, secondary outcomes, and safety). If we identify any missing or insufficient, or unclear data, we will contact primary authors to request those data.

### 2.5 Risk of bias assessment

At least 2 independent authors will assess risk of bias for each included study using Cochrane risk of bias tool, respectively. Conflicts regarding the risk of bias between 2 authors will be verified and solved by a third author if needed. It will assess each study through 7 fields, and each one is classified as low, unclear, or high risk of bias.

### 2.6 Statistical heterogeneity

Statistical heterogeneity will be checked using *I*^*2*^ statistic test, and it will be interpreted as follows: *I*^*2*^ ≤ 50% meaning low heterogeneity, and *I*^*2*^ > 50% indicating high heterogeneity.

### 2.7 Subgroup analysis

If feasible from available data, we will carry out subgroup analysis to explore the possible reasons of high heterogeneity in according to the different interventions, comparators, and outcome measurements.

### 2.8 Sensitivity analysis

We will undertake sensitivity analysis to establish stability of outcome results by eliminating high risk of bias studies.

### 2.9 Reporting bias

If sufficient trials will be entered in this study, we will check reporting bias using funnel plots.^[24]^

### 2.10 Statistical analysis

We will undertake all statistical analysis using RevMan V.5.3 software. We will estimate continuous data as mean difference or standardized mean difference and 95% confidence intervals, and dichotomous data as risk ratio and 95% confidence intervals. Whenever there is low heterogeneity, a fixed-effects model will be applied, and meta-analysis will be conducted if sufficient studies focusing on the same treatments, comparators, and outcome measurements. Whenever there is high heterogeneity, a random-effects model will be used, and a subgroup analysis will be undertaken to investigate the reasons of high heterogeneity among included studies. Additionally, we will also report outcome results as narrative summary.

### 2.11 Ethics and dissemination

This study will not need any ethical documents, because no individual patient data will be used. The results of this study will be submitted to a peer-reviewed journal for publication.

## 3 Discussion

Although a variety of studies have addressed this issue, no systematic study on this topic has been done. Thus, this study aims to assess the NE of remifentanil in CS under general anesthesia systematically. The findings of this study will help to determine that NE of remifentanil in CS under general anesthesia is effective or not. It may also provide reference evidence for both patients and clinicians, and as it may inform clinical practice guidelines.

## Author contributions

**Conceptualization:** Qi Zhang, Hong-Li Kan, Dong-Xin Wang, Dong-Mei Fu.

**Data curation:** Dong-Xin Wang, Dong-Mei Fu.

**Formal analysis:** Qi Zhang, Hong-Li Kan, Dong-Xin Wang.

**Funding acquisition:** Dong-Mei Fu.

**Investigation:** Dong-Mei Fu.

**Methodology:** Qi Zhang, Hong-Li Kan, Dong-Xin Wang.

**Project administration:** Dong-Mei Fu.

**Resources:** Qi Zhang, Hong-Li Kan, Dong-Xin Wang.

**Software:** Qi Zhang, Hong-Li Kan, Dong-Xin Wang.

**Supervision:** Dong-Mei Fu.

**Validation:** Hong-Li Kan, Dong-Mei Fu.

**Visualization:** Qi Zhang, Dong-Xin Wang, Dong-Mei Fu.

**Writing – original draft:** Qi Zhang, Dong-Xin Wang, Dong-Mei Fu.

**Writing – review and editing:** Qi Zhang, Dong-Xin Wang, Dong-Mei Fu.
